# Supplementary material for: Assessing the quality and reliability of YouTube videos as a source of information on inflammatory back pain
Source: PeerJ. 2024 Apr 11;12:e17215. doi: 10.7717/peerj.17215 (PMC11016243; doi:10.7717/peerj.17215)
Supplement: Supplemental Information 2 [file peerj-12-17215-s002.docx]

Codebook

- Country2

1: USA
2: Others

- Country4

1: USA
2: UK
3: Australia
4: Others

- Country5

1: USA
2: UK
3: Australia
4: Canada

5: Others

- Continent2
  1: America
  2: Non-America
- Source8

1: Academic
2: Physician
3: Society/Professional Organization
4: Health-related Website

5: Patient

6: News

7: Commercial

8: Non profit organizations

- Years3a

1: 2019-2023

2: 2015-2018

3: 2011-2014

- Years3b

1: 2020-2023

2: 2015-2019

3: 2011-2014

- Etiology

1:Yes

0: No

- Symtptoms

1:Yes

0: No

- Eveluation

1:Yes

0: No

- Diagnosis

1:Yes

0: No

- Treatment

1:Yes

0: No

- Differentialdiagnosis

1:Yes

0: No

- Etiology

1:Yes

0: No

- jama2

1: low reliable

2: high reliable

- jama3

1: insufficient data

2: partially sufficient data

3: completely sufficient data

- DISCERN5

1: Very Poor

2: Poor

3: Fair

4: Good

5: Excellent
